# The Effects of Leptin on Glial Cells in Neurological Diseases

**DOI:** 10.3389/fnins.2019.00828

**Published:** 2019-08-07

**Authors:** Yuki Fujita, Toshihide Yamashita

**Affiliations:** ^1^Department of Molecular Neuroscience, Graduate School of Medicine, Osaka University, Osaka, Japan; ^2^WPI Immunology Frontier Research Center, Osaka University, Osaka, Japan; ^3^Graduate School of Frontier Biosciences, Osaka University, Osaka, Japan; ^4^Department of Neuro-Medical Science, Graduate School of Medicine, Osaka University, Osaka, Japan

**Keywords:** leptin, neuron, astrocyte, microglia, oligodendrocyte

## Abstract

It is known that various endocrine modulators, including leptin and ghrelin, have neuroprotective roles in neurological diseases. Leptin is a hormone produced by adipocytes and was originally identified as a gene related to obesity in mice. The leptin receptors in the hypothalamus are the main target for the homeostatic regulation of body weight. Recent studies have demonstrated that leptin receptors are also expressed in other regions of the central nervous system (CNS), such as the hippocampus, cerebral cortex, and spinal cord. Accordingly, these studies identified the involvement of leptin in the regulation of neuronal survival and neural development. Furthermore, leptin has been shown to have neuroprotective functions in animal models of neurological diseases and demyelination. These observations also suggest that dysregulation of leptin signaling may be involved in the association between neurodegeneration and obesity. In this review, we summarize novel functions of leptin in animal models of neurodegenerative diseases. Specifically, we focus on the emerging evidence for the role of leptin in non-neuronal cells in the CNS, including astrocytes, microglia, and oligodendrocytes. Understanding leptin-mediated neuroprotective signals and molecular mechanisms underlying remyelination will be helpful to establish therapeutic strategies against neurological diseases.

## Introduction

The endocrine hormone leptin was originally identified in 1994 and is composed of 167 amino acids (aa). Leptin is encoded by the obese (*ob*) gene (Halaas et al., 1995). Leptin is predominantly produced by adipocytes and exerts its function both peripherally and centrally. This adipokine regulates food intake, metabolism, and energy homeostasis by activating receptors in the central nervous system (CNS). Peripheral leptin binds to leptin receptors in the choroid plexus and is transported across the blood–brain barrier (BBB) into the CNS. Circulating leptin levels are correlated with the body fat mass. Leptin deficiency phenotypes were first reported in mice carrying the obese (*ob*/*ob*) mutation in 1950 (Ingalls et al., 1950), which was later identified as a recessive mutation of leptin (Zhang et al., 1994). Administration of leptin completely recovers the obesity phenotype of *ob*/*ob* mice and induces anorexia and body weight loss in normal mice, suggesting that leptin negatively regulates feeding behaviors and inhibits obesity (Halaas et al., 1995; Pelleymounter et al., 1995; Rentsch et al., 1995; Weigle et al., 1995). The association of leptin and leptin receptor mutations with obesity has also been reported in humans (Montague et al., 1997; Clement et al., 1998; Strobel et al., 1998).

**FIGURE 1:**
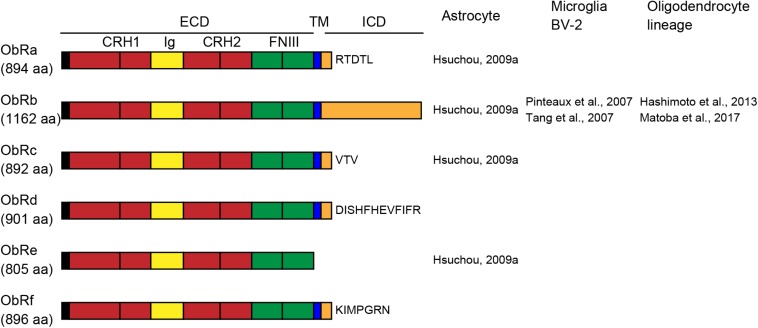
Schematic representation of leptin receptor isoforms and their domains and distribution of leptin receptor (ObR) isoforms in glial cells. The extracellular domain (ECD) of ObRs is composed of two cytokine receptor homology (CRH, red) domains, separated by an immunoglobulin-like (Ig-like, yellow) domain, followed by two membrane-proximal fibronectin type III (FN III, green) domains. ObRe lacks the transmembrane (TM, blue) and intracellular (ICD, orange) domains. The length of the intracellular domains differs among isoforms. aa, amino acids.

Leptin receptors (ObRs) are encoded by diabetes (*db*) genes and belong to the class I cytokine receptor superfamily. Six isoforms, ObRa-f, have been identified with identical N-terminal domains. Mice and rats have five ObR isoforms (ObRa-e in mice; ObRa-c, e, and f in rats), whereas humans have four (ObRa, b, c, and e) (Cioffi et al., 1996; Ahima and Flier, 2000; Wauman and Tavernier, 2011). All ObR isoforms except the ObRe isoform are expressed at the plasma membrane. The extracellular domain of all ObRs consists of two cytokine receptor homology (CRH) domains separated by an immunoglobulin-like (Ig-like) domain, followed by two membrane-proximal fibronectin type III (FN III) domains (Figure 1). The membrane-proximal CRH2 domain is essential for leptin binding (Fong et al., 1998). The two FN III domains are required for receptor activation and transduction of leptin signaling (Prokop et al., 2012). The length of the intracellular domain differs among isoforms. The long isoform of the mouse ObRb has a 302-aa intracellular domain (Uniprot^1^) and fully transduces leptin receptor signaling. The short isoforms (ObRa, c, d, and f) have intracellular domains of 30–40 aa and transduce only some of the ObRb-induced signals. Because ObRe has no transmembrane region, it is considered to serve as an antagonist for leptin signaling and to mediate leptin transport across the BBB (Tu et al., 2008), as well as to support the stability and availability of circulating leptin (Basharat et al., 2014). Leptin binding to ObRb increases Janus tyrosine kinase 2 (JAK2) phosphorylation, leading to the activation of various intracellular signaling molecules, including signal transducer and activator of transcription 3 (STAT3), mitogen-activated protein kinase (MAPK), and phosphatidylinositol 3-kinase (PI3K) (Chen et al., 2015; Fazolini et al., 2015).

Because leptin receptors are expressed not only in the hypothalamus, but also in other areas of the brain, including the cerebral cortex, hippocampus, ventral tegmental area, substantia nigra, medulla, and cerebellum, the role of leptin extends beyond its usual hypothalamic function. Leptin signaling is thought to play a role in processes such as learning, memory, and neuroprotection following neurodegeneration. In this review, we discuss the neuroprotective effects of leptin in CNS diseases. Accumulating studies have reported that leptin functions in non-neuronal cells in the CNS. Here, we also focus on the current understanding of glial leptin functions and signaling mechanisms.

## Expression of Leptin and Leptin Receptors

Leptin is produced mainly in adipose tissue, but is also produced in organs such as the stomach, mammary glands, placenta, skeletal muscle, heart, kidney, and brain (Bado et al., 1998; Smith-Kirwin et al., 1998; Morash et al., 1999; Wilkinson et al., 2000; Lin et al., 2011; Shan and Yeo, 2011; Tessier et al., 2013; Wang et al., 2014; Feijoo-Bandin et al., 2015). These findings suggest that leptin has many diverse functions. Accordingly, disturbance of leptin signaling affects various systems, including the cardiovascular, immune, reproductive, and nervous systems (Donato et al., 2011; Procaccini et al., 2012).

The long isoform of the leptin receptor ObRb is expressed in the hypothalamic nuclei, including the arcuate (ARC) nucleus, the dorsomedial (DMH) nucleus, the paraventricular (PVN) nucleus, the ventromedial hypothalamic (VMH) nucleus, and the lateral hypothalamic (LH) nucleus (Mercer et al., 1996; Schwartz et al., 1996; Friedman and Halaas, 1998; Yi et al., 2013). The expression of leptin in these areas implicates that leptin functions as a metabolic hormone, and many other reviews have already summarized the role of leptin in the hypothalamus (Ahima and Flier, 2000; Margetic et al., 2002; Leinninger, 2011). The expression of other ObR isoforms has also been found in extrahypothalamic nuclei in the cerebral cortex, hippocampus, ventral tegmental area, substantia nigra, medulla, and cerebellum (Elmquist et al., 1998; Figlewicz et al., 2003; Mutze et al., 2006). More specifically, ObRb is expressed in the neurogenic niche in the dentate gyrus of the adult hippocampus (Garza et al., 2008), suggesting that leptin signaling may play a role in the regulation of neurogenesis. The short isoforms ObRa and ObRc, but not ObRb, are abundantly expressed in the cerebral microvessels composing the BBB, suggesting that these receptors may be associated with leptin transport (Tartaglia et al., 1995; Bjorbaek et al., 1997; Golden et al., 1997).

**FIGURE 2:**
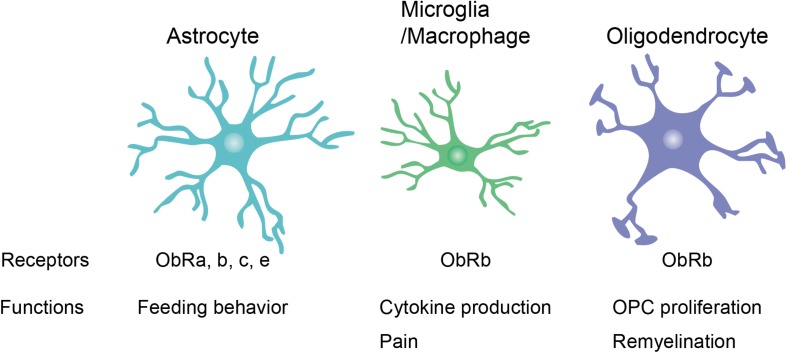
Leptin receptor (ObR) expression and function in glial cells. Reported expression of ObR isoforms and functions of ObRs in glial cells are summarized. OPC, oligodendrocyte precursor cell.

Leptin receptor expression has been observed in various types of neurons, including glutamatergic, GABAergic, and dopaminergic neurons (Figlewicz et al., 2003; Vong et al., 2011; Xu et al., 2013; Yi et al., 2013). Therefore, neurons have been well-studied as the primary leptin targets in the CNS. However, leptin receptor mRNA expression has also been found in glial cells in normal CNS tissue such as the mouse spinal cord and the rat hypothalamus (Hsuchou et al., 2009b; Figure 2). The following section “Leptin Signaling in Glial Cells” focuses on the expression and function of leptin and leptin receptors in glial cells.

## Neuroprotective Roles of Leptin

Besides the inhibition of food intake, leptin signaling helps regulate neurogenesis, synaptogenesis, and neuronal excitability (Bouret, 2010; Paz-Filho et al., 2010; Arnoldussen et al., 2014). ObRs are highly expressed in synaptic regions of cultured hippocampal neurons (Shanley et al., 2002). Consistent with this synaptic expression pattern, leptin potently regulates hippocampal synaptic function (McGregor and Harvey, 2018). Furthermore, leptin regulates neurogenesis during the development of the CNS. These functions suggest that leptin may have protective functions in neurological diseases and insults. The association of increased leptin levels in obesity and increased risk of neurodegenerative diseases in obese individuals has been widely discussed (Forny-Germano et al., 2018; Lloret et al., 2019). In animal disease models, neurotoxin 1-methyl-4-phenyl-1, 2, 4, 5-tetrahydropyridine (MPTP)- or 6-hydroxydopamine (6-OHDA)-induced Parkinson’s disease (PD) model show greater neurodegeneration with obesity compared to littermates fed a normal diet (Choi et al., 2005; Morris et al., 2010). Midlife adiposity is associated with the risk of PD (Abbott et al., 2002; Procaccini et al., 2016). Furthermore, midlife obesity is also linked to a higher risk of developing cognitive disorders, including Alzheimer’s disease (Kivipelto et al., 2005; Rosengren et al., 2005; Whitmer et al., 2005). It has been reported that plasma levels of leptin are decreased in PD and Huntington’s disease (Pratley et al., 2000; Evidente et al., 2001; Popovic et al., 2004). Leptin also shows the protective effects on pediatric neurological diseases. The human seizures frequently occur early in life. Zn^2+^ is one of the most abundant divalent metal ions in the CNS (Chimienti et al., 2003; Devirgiliis et al., 2007), and zinc transporters show dynamic expression pattern in animal seizure model (Ni et al., 2010, 2011a, b). Zinc transporters ZnT3, ZnT4, and ZIP7 are upregulated in cultured HT22 cells and flurothyl-induced recurrent neonatal seizures, and these altered expressions were reversed by leptin treatment (Jin et al., 2018; Li et al., 2018). Leptin suppresses aberrant mossy fiber sprouting and hippocampal-related cognitive impairment in rat developmental seizure models (Li et al., 2018; Ni et al., 2018). Thus, there is emerging evidence for the neuroprotective roles of leptin in neurological diseases. As there are systematic reviews on the neuroprotective effects of leptin in CNS diseases (Signore et al., 2008; Tang, 2008; Davis et al., 2014; Li et al., 2016), here we focus on the role of leptin signaling in non-neuronal cells in the CNS, including astrocytes, microglia, and oligodendrocytes.

## Leptin Signaling in Glial Cells

### The Role of Leptin in Astrocytes

Astrocytes are glial cells involved in homeostatic control and neuroprotection (Verkhratsky and Nedergaard, 2018). Astrocytes express several ObR isoforms, including ObRa, ObRb, ObRc, and ObRe (Hsuchou et al., 2009a). Primary cultured astrocytes prepared from the mouse hypothalamus mainly express the isoforms ObRa and ObRb. In addition to these isoforms, ObRc and ObRe are expressed in cells prepared from the mouse hippocampus. ObR mRNA expression has also been detected in astrocytes in the rat hypothalamus via fluorescent *in situ* hybridization and immunohistochemistry (Hsuchou et al., 2009b). ObRs are expressed by glial fibrillary acidic protein (GFAP)-positive cells in the hypothalamus. There have been reports on regional differences of the cell types expressing ObRs. In the ARC, ObR immunofluorescence is predominantly observed in neurons, whereas it is mainly detected in cells with a morphology resembling astrocytes in the DMH hypothalamus (Pan et al., 2008). Additionally, in the ARC, a specialized subpopulation of astrocytes that shows intense immunoreactivity for brain fatty acid-binding protein, which is considered to be involved in the regulation of feeding, is in close proximity to leptin-sensitive neurons (Young, 2002).

It has been reported that upregulation of astrocytic ObRs is involved in adult-onset obesity (Pan and Kastin, 2007; Pan et al., 2008; Hsuchou et al., 2009a). The agouti viable yellow (*A*^vy^) mouse has a spontaneous mutation consisting of an insertion of a retrotransposon in the promoter region of the gene encoding the agouti signaling protein, which induces ectopic overexpression of the agouti-related protein (AgRP), leading to antagonism of the melanocortin receptors (MCRs). This produces two prominent phenotypes in the *A*^vy^ mouse. The first is dysregulation of MCR-1 signaling in the skin, which increases pheomelanin synthesis and results in the agouti coat. The second phenotype results from defects in MCR-4 signaling, which causes hyperphagia and obesity (Dickies, 1962; Wolff, 1965; Wolff et al., 1986; Shimizu et al., 1989; Duhl et al., 1994; Wolff and Whittaker, 2005). The *A*^vy^ mouse shows increased ObR expression in astrocytes and increased ObRb mRNA in microvessels, which may play crucial roles in the reduced apparent influx of leptin from the blood to the brain (Pan and Kastin, 2007). Furthermore, inhibition of astrocytes in *A*^vy^ mice via intracerebroventricular treatment with the astrocyte-specific metabolic inhibitor fluorocitrate reduces the uptake of fluorescently labeled leptin into astrocytes and attenuates leptin-induced phosphorylation of STAT3, whereas neuronal uptake in the ARC and DMH hypothalamus is increased (Pan et al., 2011). These observations suggest opposite roles of astrocytic leptin signaling in neurons of the *A*^vy^ mouse. ObR-positive astrocytes are also increased in diet-induced obesity in adult B6 mice (Hsuchou et al., 2009a).

Leptin has also been reported to affect astrocyte morphology and synaptic protein levels in the hypothalamus, and it can rapidly induce synaptic changes in feeding circuits of *ob*/*ob* mice (Pinto et al., 2004; Horvath et al., 2010; Garcia-Caceres et al., 2011). Chronic intracerebroventricular leptin treatment modulates the astrocyte-specific glucose transporters (GLUT)-2, GLUT-3, and glutamate transporter (GLAST) (Fuente-Martin et al., 2012) in the hypothalamus, suggesting that leptin signaling in astrocytes may influence glutamate levels in the synaptic cleft, as well as energy homeostasis. Conditional deletion of astrocytic leptin receptors using Cre-loxP systems (GFAP-Cre; leptin receptor flox/flox) alters the astrocyte morphology and increases the number of synapses on proopiomelanocortin (POMC) and AgRP neurons, which are involved in the hypothalamic feeding control (Kim et al., 2014). Deletion of ObR in astrocytes diminishes leptin-induced anorexia and enhances fasting- or ghrelin-induced hyperphagia. These observations suggest that astrocytic leptin signaling actively controls feeding behavior.

### The Role of Leptin in Microglia and Macrophages

The murine BV-2 cell line and rat microglia express both the long and short isoforms of the leptin receptor (Pinteaux et al., 2007; Tang et al., 2007). Semi-quantitative RT-PCR showed that ObRb mRNA expression is elevated in microglia compared to that in astrocytes and neurons, whereas ObRa mRNA expression levels are higher in astrocytes and microglia compared to that in neurons (Pinteaux et al., 2007).

Leptin has been reported to have an effect on cytokine production in microglia/macrophages (Pinteaux et al., 2007; Tang et al., 2007; Lafrance et al., 2010). Leptin activates ObRb in rat primary microglial cells and induces interleukin (IL)-1β production via STAT3 activation (Pinteaux et al., 2007). In primary hypothalamic microglia, leptin treatment induces IL-1β and tumor necrosis factor-α (TNF-α) but not ionized calcium binding adaptor molecule 1 (Iba1) expression, and disruption of leptin signaling in *ob*/*ob* or *db*/*db* mice alters the expression of genes involved in microglial functions (Gao et al., 2014). Pretreatment of rat brain microglial cells with leptin increases the lipopolysaccharide (LPS)-induced expression of IL-1β, TNF-α, and chemokines such as cytokine-induced neutrophil chemoattractant-1 (CINC-1) and macrophage inflammatory protein-2 (MIP-2) (Lafrance et al., 2010). Leptin treatment has also been shown to increase IL-6 production in BV-2 cells via the leptin receptor/IRS-1/PI3K/Akt/NF-κB/p300 signaling pathway, as leptin-induced IL-6 production is diminished by inhibitors for these signaling molecules (Tang et al., 2007). Plasma leptin levels are positively correlated with the number of Iba1-positive cells in the ARC of mice fed with a high saturated fat diet, raising the possibility that overnutrition-dependent inflammation is associated with leptin signaling in microglia (Andre et al., 2017). Furthermore, deletion of the leptin receptor in myeloid cells, including microglia and macrophages, by crossing Cx3cr1-Cre mice with ObR-loxP mice disrupts hypothalamic neuronal circuits, that regulate metabolism, and increases body weight (Gao et al., 2018). In this mouse, the number of POMC neurons in the ARC and alpha-melanocyte-stimulating hormone (α-MSH) projections from the ARC to the PVN are decreased, concurrent with the presence of less ramified microglia with impaired phagocytic capacity in the PVN. These observations suggest that the microglial ObR mediates, at least in part, the action of leptin in regulating inflammation.

Leptin has been shown to be involved in the activation of microglia and macrophages in the context of neuropathic pain. Microglia have been known to play a critical role in mechanisms of neuropathic pain (Coull et al., 2005; Koizumi et al., 2007; Beggs and Salter, 2010; Yasui et al., 2014; Inoue and Tsuda, 2018). Microglial activation in the spinal cord is involved in nerve injury-induced pain hypersensitivity, which characterizes neuropathic pain. Intrathecal administration of a leptin antagonist prevents the development of thermal hyperalgesia and mechanical allodynia in the rat chronic constriction injury (CCI) model (Lim et al., 2009). Leptin-deficiency in mice also abolishes CCI-induced neuropathic pain behaviors. Expression of both leptin and ObRb is increased in the ipsilateral dorsal horn of the spinal cord after injury. In *in vitro* organotypic lumbar spinal cord cultures, leptin treatment increases OX-42 (CD11b/c)-positive cells and upregulates IL-1β, as well as the NR1 subunit of the NMDA receptor, via the JAK/STAT pathway, suggesting that microglia are a possible source of IL-1β. In the partial sciatic nerve ligation (PSL, Seltzer model) model (Seltzer et al., 1990), leptin expression is increased in adipocytes and in the epineurium of the injured sciatic nerve. However, PSL-induced tactile allodynia does not develop in the leptin-deficient *ob/ob* mouse (Maeda et al., 2009). In this model, macrophages are recruited to the perineurium of the sciatic nerve and express the leptin receptor, and leptin stimulates macrophages through the JAK–STAT pathway. Treating the macrophage cell line J774A.1 with leptin induces mRNA expression of matrix metalloproteinase-9 (MMP-9) and inducible nitric oxide synthase (iNOS), which are molecules known to underlie allodynia development via phosphorylated STAT3. These findings suggest that leptin may be associated with the development of neuropathic pain via activation of macrophages.

By contrast, it has been reported that leptin reduces microglial activation and contributes to functional recovery in the rat spinal cord injury (SCI) model (Fernandez-Martos et al., 2012). Both leptin and ObRb mRNA expression levels are increased after SCI. Intraparenchymal injection of leptin enhances functional motor recovery and prevents neuropathic pain after SCI. Leptin administration decreases the expression of inflammatory genes and decreases the area of Iba1-positive microglia/macrophages after SCI. Thus, leptin-controlled inflammatory modulation in microglia shows diverse effects and depends on the animal model. Further investigations using cell-type-specific deletions of leptin and leptin receptors will be helpful in clarifying the role of leptin signaling in microglia.

### The Role of Leptin in Oligodendrocytes

Expression of ObRs is also detected in oligodendrocyte lineage cells. ObRb is expressed in NG-2 positive oligodendrocyte precursor cells (OPCs) but not in A2B5-positive oligodendrocyte progenitors in the postnatal day (P)7 mouse cerebrum. By P14, ObRb expression is detected in O4-positive mature oligodendrocytes and OPCs (Hashimoto et al., 2013), indicating that ObRb expression depends on the differentiation status of the cells. Embryonic day (E)18 *ob/ob* mice show increased numbers of OPCs compared to wild-type mice, suggesting that leptin can attenuate oligodendrocyte development in the mouse embryonic cerebral cortex (Udagawa et al., 2006). By contrast, myelin basic protein (MBP) mRNA expression is at P14 and P28 lower in *ob*/*ob* mice than in wild-type mice, and leptin-treated *ob/ob* mice exhibit increased myelination, suggesting that leptin regulates differentiation and/or myelination of oligodendrocytes (Hashimoto et al., 2013). Obesity also seems to be associated with oligodendrocyte maturation and myelination. High fat diet-induced adult-onset obesity may reduce myelin thickness and inhibit oligodendrocyte maturation. OPC differentiation following focal white matter stroke is impaired in obese mice, while their platelet-derived growth factor receptor alpha (PDGFRα)-positive OPC response in the early phase after stroke is exaggerated.

Immunohistochemistry in spinal cord tissue showed that ObRb protein expression is observed in PDGFRα-positive OPCs, GFAP-positive astrocytes, and NeuN-positive neurons (Matoba et al., 2017). Demyelination induced by lysophosphatidylcholine (LPC) injection does not affect ObRb expression in these CNS cells. However, intrathecal administration of leptin-neutralizing antibodies decreases bromodeoxyuridine (BrdU)- and PDGFRα-double-positive proliferating OPCs after LPC injection and induces a reduction in myelin formation, as characterized by a decrease in MBP-positive area, suggesting that leptin signaling supports an increase in OPC proliferation and remyelination in LPC-induced demyelination. In the SCI model, immunohistochemistry showed that ObRb expression is increased in APC-positive oligodendrocytes and, to a lesser extent, in GFAP-positive astrocytes, OX-42-positive microglia/macrophages, and NeuN-positive neurons 24 h after injury (Fernandez-Martos et al., 2012). Leptin administration also increases myelin preservation caudal to the lesion epicenter. Although whether expression of ObRb increases differs from the injury model, leptin appears to be increased in the spinal cord after both demyelination and injury. Thus, these studies suggest that leptin signaling in oligodendrocyte lineage cells may contribute to cell and tissue repair after injury or other pathological conditions in the CNS.

## Conclusion

Growing evidence from basic research supports that glial functions control the metabolism (Garcia-Caceres et al., 2019). Beyond its roles in food intake and energy homeostasis, leptin has been found to have protective roles in the CNS. Accumulating results from studies on the neuroprotective functions of leptin signaling in animal models of neurodegeneration and observations in individuals with neurodegenerative disorders support the hypothesis that leptin and leptin receptors are potential therapeutic targets in neurodegenerative diseases. However, there might be some limitations to the therapeutic use of leptin. For example, glial leptin can have both beneficial and detrimental effects depending on the cell type and the context of the neurological disease or insult. To enhance the neuroprotective functions and to prevent the detrimental processes and unwanted effects, it will be important to further our understanding of leptin signaling in glial cells.

## Author Contributions

All authors listed have made a substantial, direct and intellectual contribution to the work, and approved it for publication.

## Conflict of Interest Statement

The authors declare that the research was conducted in the absence of any commercial or financial relationships that could be construed as a potential conflict of interest.

## Footnotes

1 www.uniprot.org/uniprot/P48356
